# Mr. Marson, on the Delivery of the Placenta

**Published:** 1803-11-01

**Authors:** W. Marson

**Affiliations:** Member of the College of Surgeons. Worksop


### *To the Editors of the Medical and Physical Journal.*

Gentlemen,

As I have long been convinced of the advantage of considerable pressure for the more ready deliverance of the placenta, and of its being otherwise of good effect, I send you the following observations on that subject.

Let us suppose the woman to be delivered. As soon as the child is born, and convincing myself that it breathes, and before tying the funis, I apply my left hand to the sacrum, and the right hand (both exterior to the clothes) to the parietes of the abdomen on the region of the dilated uterus. I then make a slight pressure with both hands, and increase it gradually till I have exerted sufficient force for nearly two minutes, unless interrupted by the pain (indicating a contraction of the uterus) as Dr. Hunter used to describe it. I frequently relieve my right hand during the pressure, with the idea of forming a kneading motion, as will be described below, but I always keep up the force I had gradually carried it to. I then secure the navel string and give the child to the nurse, when I again apply to the funis, and find the placenta waiting for me either at the os externum, in the vagina, or so as to be brought away directly or in a minute or two afterwards; if it should not, I apply pressure as before, and the business is generally compleated; but I should repeat it again and again, if necessary.

If we consult the different authors and lecturers on midwifery, we find a gentle pressure recommended by some of them on the parietes of the abdomen as soon as the child is born, "to press the air from the uterus, and to assist it in its contraction." But they do not, I observe, assign any reason why this pressure should be so slight; yet where it is necessary to introduce the hand into the uterus to turn the child, the attendant is told to press hard with both her hands on the abdomen, to prevent the uterus rolling; and this without a seeming notion of the pressure doing any harm, because it is not even mentioned afterwards, but they speak of a gentle pressure, as above mentioned, as if they conceived a stronger one would be injurious, or perhaps they may think a slighter pressure sufficient, as corresponding with what they intend to be effected by it; but as it does not by any means agree with my idea, I shall beg leave to assign my reasons for a continued strong pressure.

In labour in general there is more or less of faintness comes on when the child is born; this is ascribed to the fatigue of labour, to the discharge, and to the sudden taking off of tension. The latter I consider as the chief cause; and if so, pressure must be as advantageous as in the operation of the paracentesis, which no practitioner, I believe, would neglect.

The uterus, when the child is born, being left in a state of flaccidity, the muscular fibres I have imagined to be fatigued with their exertion, less disposed to contract during the faintness; that they could not be so soon excited to contract, having lost the distension, the irritating cause; that if it is the first child, the overjoy of the mother leads the mind quite astray from any thing further than its birth to be accomplished, and though it (the mind) may not have any voluntary power on the uterus: I conceive then it is our duty to promote its action, and this by means of pressure, which will assist it in its contraction; but as there must be frequently great atony and want of irritability, a gentle pressure of short continuance is not so likely to fulfil the intention as that I speak of.

Admitting the placenta to be urged forward by pressure, it is very clear that the hour glass contraction, as it is called, when this is practised, cannot take place, the muscular fibres of the fundus uteri not contracting, and those of the middle or sides of the uterus doing so, the pressure on the bulk of the placenta would overcome the circular contraction; I have thought so much of this, that if ever I was called to a case of retention of placenta, and it had been previously ascertained that there had been an hour glass contraction in former labours, I would endeavour to surmount it by pressure, rather than the introduction of the hand.

The unpleasant case of flooding after the child is born, will be less liable to be fatal where considerable pressure is used, for provided we should not think it right to immediately ately extract the placenta, pressure would approximate the sides of the uterus, and close these patulent vessels. If the placenta is extracted, still pressure is advisable, as the uterus may remain in a state of flaccidity from its inertness or inirritability.

I particularly recommend this to the consideration of your numerous readers; the application of considerable pressure in most cases of uterine hæmorrhage, I am persuaded, is calculated to be of advantage, and I am positive can do no harm. The matter, I conceive, speaks for itself, or I would encroach more on your time and patience to illustrate and enforce it. I am, &c.

W. MARSON,

Member of the College of Surgeons.

*Worksop, Notts, Sept. 20, 1803.*

